# CircRNA-UCK2 Increased TET1 Inhibits Proliferation and Invasion of Prostate Cancer Cells Via Sponge MiRNA-767-5p

**DOI:** 10.1515/med-2019-0097

**Published:** 2019-11-20

**Authors:** Zhendong Xiang, Chengdang Xu, Gang Wu, Bo Liu, Denglong Wu

**Affiliations:** 1 Department of Urology, Tongji Hospital, Tongji University School of Medicine, Shanghai 200092, China; 2 Department of Urology, The People’s Hospital of China Three Gorges University, The First People’s Hospital of Yichang, Yichang 443003, China

**Keywords:** EnzR PCa, circUCK2, miR-767-5p, TET1, invasion, proliferation

## Abstract

A majority of the patients with advanced prostate cancer initially respond to androgen deprivation therapy and enzalutamide therapy, but eventually enter the castration-resistant prostate cancer (CRPC) phase. Some studies have shown that the activation of other signalling pathways in CRPC cells replaces the function of the androgen receptor, as well as promotes cell metastasis and progression. However, the mechanisms underlying this side effect remain unclear. The present study aims to explore the continued progression of cells after enzalutamide resistance. Low expression of circRNA-UCK2 (circUCK2) was detected in enzalutamide-resistant (EnzR) cells. Moreover, miR-767-5p was found to be resistant to EnzR cells when the level of circUCK2 is increased. The decrease in free miR-767-5p increases the expression of TET1 protein through the post-transcriptional regulation of mRNA, thereby inhibiting cell invasion and proliferation. Knocking down circUCK2 in enzalutamide-sensitive cells reduces the concentration of TET1, thereby increasing cell invasion and proliferation. A preclinical study using in vivo mouse models also showed that a high expression of circUCK2 inhibited the EnzR cell growth. Thus, this study might aid in developing a novel therapy to better suppress the CRPC progression.

## 1 Introduction

Prostate cancer (PCa) is the most common cancer and the third leading cause of deaths in men [1]. Androgen deprivation therapy (ADT), achieved through surgery and chemotherapy, is the main therapy for advanced PCa. Enzalutamide (Enz) is a second-generation endocrine therapy drug; it can competitively block the transfer of androgen receptors (ARs), recruit the co-activators, effectuate binding with the AR DNA, and activate the AR target genes [2].

Reportedly, long-term anti-androgen therapy with Enz increases the metastatic ability of castration-resistant PCa [3, 4, 5]. The inhibition of the AR signalling enhances the growth factor signalling pathway that replaces the function of AR[6, 7, 8]. Moreover, the long-term inhibition of AR promotes the expression of AR-v7, the activation of the RANK signalling pathway, and NCoA2 and the TGFβ/Smad3/MMP9 signalling pathway to maintain and promote the growth and metastasis of cells [5, 9]. Enz targets the ARs that are vital intracellular transcription factors, capable of regulating multiple signalling pathways. The increase of cell invasiveness and tumour development is a sophisticated process and needs to be addressed from several perspectives.

Circular RNA (circRNA) is a class of circular non-coding RNA molecules without a 5’ end and a 3’ end [10]. On the one hand, it can compete with complementary complements of introns to affect the expression of homologous genes and protein translation[11]; on the other hand, it can adsorb miRNAs, reduce the number of free miRNAs, and inhibit its function [12]. Recent studies have suggested that circRNA can stabilize miRNAs and promote the function of target miRNAs. In our previous studies, it was also found that circRNA can affect AR variant-7 to regulate prostate cancer progression [13].

The purpose of the present study was to investigate the cause of increased cell invasiveness and further development of PCa enzalutamide resistance (EnzR) with respect to circRNAs. We found that circUCK2 significantly decreased the expression of EnzR-PCa cells, and the miRNA sponge effect reduced the expression of TET1, thereby increasing cell proliferation and invasion.

## 2 Material and Methods

### 2.1 Cell culture

The human PCa cell line C4-2 was obtained from the American Type Culture Collection (ATCC, Rockville, MD, USA) and cultured in RPMI-1640 supplemented with 10% fetal bovine serum (FBS) and 1% penicillin-streptomycin, in a 5% (v/v) CO2 humidified incubator at 37°C. EnzR-C4-2 cells were generated by culturing the EnzS-C4-2 cells in the presence of increasing Enz concentrations from 10–40 μM (every 20 days) for 3 months. All EnzR cells were maintained with 10 µM Enz.

### 2.2 MTT assay

Cell proliferation was evaluated by MTT assay as reported work [14]. The cells were seeded in 96-well plate at a density of 1000 cells/well, adhered overnight, and cultured at 37°C, 5% CO2 for days 2, 4, and 6. Then, the cells were harvested and the cell number was calculated using an MTT reagent. DMSO was used as the control.

### 2.3 Tests of RNase R resistance

The total RNAs were isolated from cells using TRIzol (Invitrogen), followed by PureLink purification of the aqueous phase (Life Technologies). An equivalent of 1 µg of total RNA was treated with 0 (mock treatment) or 20 U of RNase R (Epicenter) in 1X RNase R buffer in a 10 µL reaction containing 1U/µL murine ribonuclease inhibitor (New England Biolabs). The reaction was incubated at 37°C for 1 h, followed by the addition of 1 µL of 10 mM dNTP, 1 µL of 1 mM EDTA, and 1 µL of 100 µM random hexamers. The RNA was denatured at 65°C for 5 min and placed on ice. Then, 4 µL of buffer (250 mM Tris-HCl pH 8, 125 mM KCl, 15 mM MgCl2), 1 µL murine ribonuclease inhibitor (40 U/ µL), and 1 µL Superscript III (Life Technologies) constituted the reaction for the synthesis of cDNA at 25°C for 10 min, 50°C 50 min, 55°C 10 min, 85°C 5 min, then held at 4°C. Subsequently, 1 µL cDNA reaction was adopted as the template for qPCR.

### 2.4 Lentiviral expression plasmids and virus production

The plasmids pLCDH-circUCK2, Plv-miR-767-5p, pLKO.1-TET1, the psPAX2 packaging plasmid, and pMD2.G envelope plasmid, were transfected into HEK-293T cells using the standard calcium chloride transfection method for 48 h to obtain the lentivirus soup. These soups were collected, concentrated by density gradient centrifugation, and used immediately or preserved at -80°C for later use.

### 2.5 RNA extraction and quantitative real-time PCR (qRT-PCR) analysis

The total RNA was extracted using TRIzol reagent. An equivalent of 1 µg of total RNA was subjected to reversed transcription into cDNA using PrimeScript™ RT reagent Kit(Takara) and Mir-X™ miRNA qRT-PCR SYBR® Kit (Takara). The mRNA and miRNA expression of the target gene was determined by qRT-PCR conducted on ABI-7900 system with SYBR Green (Takara). The expression of a target gene was normalized to that of *GAPDH or U6*.

### 2.6 Western Blot Analysis

We follow the method with reported work[15]. Cells were lysed in RIPA buffer, and an equivalent of 40 µg total protein was separated on 10% SDS-PAGE and transferred onto 0.45 μm polyvinylidene fluoride (PVDF) membranes (Millipore). The membranes were blocked and probed with specific primary antibodies, followed by incubation with HRP-conjugated secondary antibodies. The immunoreactive bands were visualized using the ECL system (Thermo Fisher Scientific).

### 2.7 Transwell invasion assay

The cell invasion capacity was assessed by a Matrigel invasion assay by coating the Matrigel on 8.0-μm filter membranes. An equivalent of 1 × 105 cells in 150 μL serumfree medium was plated onto each filter, with 750 μL of 10% fetal bovine serum-containing medium placed in the lower chamber. The system was incubated for 24 h in a humidified tissue culture incubator at 37°C in a 5% CO2 atmosphere. After 24 h, the membranes were fixed with 4% paraformaldehyde on both sides, washed with phosphate buffer saline (PBS), and stained with crystal violet. The cells on the upper surface of the filters were removed with cotton swabs, and those that invaded the lower surface of the filter were counted under the microscope. The relative invasion was determined by setting the number of invading cells in the ethanol-treated group as one.

### 2.8 Pull-down assay

The cells were harvested and lysed in RIPA lysis buffer. The supernatant was incubated with 500 pM of anti-sense oligos supplemented with RNase inhibitor overnight at 4°C. Subsequently, 10u µL Streptavidin Agarose beads were added and to the cells, and the mixture homogenized for 2 h at 4°C. Then, the streptavidin Agarose beads were incubated with the supernatant for 2 h. The complex was centrifuged at a speed of 3000 rpm for 10 mins, following which, the beads were washed five times with RIPA lysis buffer. The RNA was extracted using TRIzol according to the manufacturer’s protocol and subjected to RT-PCR analysis.

### 2.9 In vivo studies

7-week-old NOD/SCID mice were purchased from Slac Laboratory Animal and divided into two groups (9 mice/ group) for injection of 1×106 EnzR-C4-2 cells and pre-cultured as follows: 1: pLCDH; 2: oe-circUCK2. A total of 1x106 EnzR-C4-2 cells were mixed with Matrigel (1:1) and transplanted into the prostate of the male NOD/SCID mice. Next, the mice were injected with 20mg/kg enzalutamide intravenously every 2 days. Tumour development was monitored by non-invasive In Vivo Fluorescent Imager (IVIS Spectrum, Caliper Life Sciences, Cleveland, USA) once per week. The mice were sacrificed after 8 weeks, and any metastatic tumours were removed for subsequent analysis. This study revealed in specific pathogen-free (SPF) conditions in strict accordance with the Ethics Committee of Tongji Hospital.

### 2.10 Immunohistochemistry (IHC) staining

Mouse tissues were fixed in 10% (v/v) formaldehyde in PBS and embedded in paraffin. The samples were sliced into 5-μm-thick sections, dehydrated with xylene, and used for IHC staining with a specific primary antibody against TET1. To enhance antigen exposure, the slides were treated with 1X EDTA at 98°C for 10 min for antigen retrieval. The slices were incubated with endogenous peroxidase blocking solution, followed by incubation with the primary antibody TET1 overnight at 4 ºC. After rinsing with the phosphate buffer saline, the slides were incubated with biotinylated secondary antibody for 1 h at room temperature, washed, and incubated with enzyme conjugated horseradish peroxidase (HRP)-streptavidin. Freshly prepared DAB (Zymed, San Francisco, CA, USA) was used as a substrate to detect the HRP. Finally, slides were counterstained with hematoxylin and mounted with aqueous mounting media. The number of positive cells was calculated as the number of immune-positive cells × 100% / the total number of cells/field in 10 random fields at 400X magnification.

### 2.11 Statistical analysis

All statistical analyses were carried out using SPSS 19.0 (SPSS Inc, Chicago, IL, USA). Data were presented as the mean ± SD or SEM. The differences in the mean values between two groups were analyzed by two-tailed Student’s t-test, and the mean values of more than two groups were compared using one-way ANOVA. *p* ≤ 0.05 was considered statistically significant.

## 3 Results

### 3.1 CircUCK2 decrease EnzR cell proliferation and invasion

**Figure 1:**
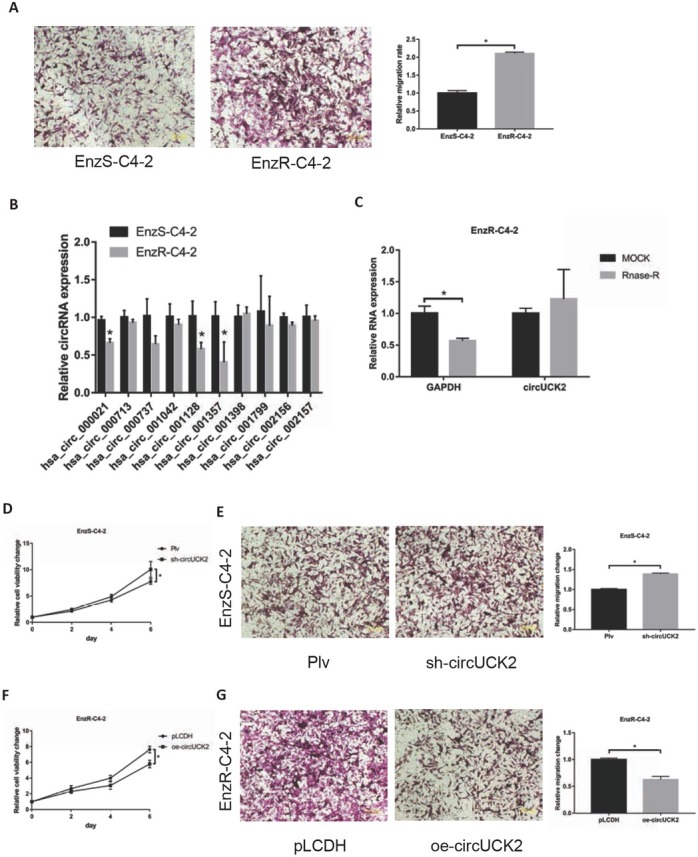
CircUCK2 decrease EnzR cell proliferation and invasion. (A) Transwell invasion assay was performed to show the different invasion capacity in Enzalutamide-sensitive C4-2 cell line (EnzS-C4-2) and Enzalutamide-resistance C4-2 cell line (EnzR-C4-2). (B) Ten circRNAs related to cell proliferation and invasion were screened from the literature and expressed differently in the comparator between EnzS cells and EnzR cells. (C) The RNase-R assay was used to determine the sensitivity of circUCK2 to RNase digestion. (D-E) Knocking down circUCK2 in EnzS-C4-2 cells leads to increase cell proliferation and invasion. (F-G) The overexpression of circUCK2 in EnzR-C4-2 cells leads to decreased cell proliferation and invasion. The data shown represent the mean of three independent experiments. *p < 0.05 by Student’s t-test for two groups or ANOVA for more than two groups.

PCa eventually progressed to castration-resistant PCa after antiandrogen therapy[16], and enzalutamide only extended the patient’s survival an extra 4.8 months[17]. The Transwell invasion assay showed that EnzR-C4-2 cells were more invasive than EnzS-C4-2 cells (Fig. 1A). In our previous study, circRNAs were found to play a role in the development of PCa. Based on the previous results, ten genes related to tumour growth and invasion were identified as candidates through prediction literature research and database. Hsa_circ-001128 and hsa_circ_001357 (circUCK2) were found to be significantly decreased in EnzR-C4-2 cells compared to EnzS-C4-2 cells (Fig. 1B). CircUCK2 was insensitive to RNase-R, which was confirmed by RNase-R assay. (Fig. 1C). We found that circUCK2 can change the proliferation and invasion abilities of PCa cells, while hsa_circ-001128 cannot. EnzS-C4-2 and circUCK2 knockdown can accelerate cell proliferation and invasion (Fig. 1D-E). After the overexpression of circUCK2, cell proliferation slowed down, and invasion decreased (Fig. 1F-G).

Taken together, the results from Fig. 1A–G suggested that circUCK2 can suppress the growth of EnzR PCa cells.

### 3.2 Mechanism dissection of how circUCK2 can suppress the PCa cell growth: via sponge miR-767-5p

**Figure 2:**
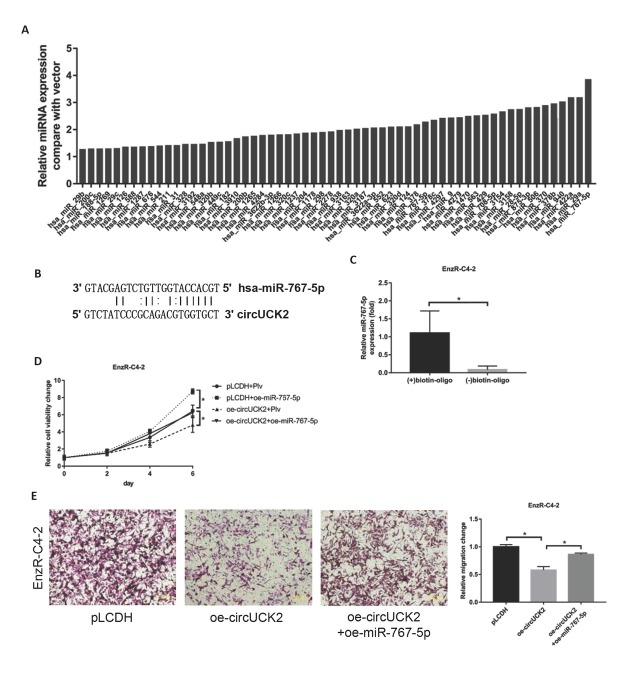
Mechanism dissection of how circUCK2 can suppress PCa cell growth via sponge the miR-767-5p. (A) The expression of miRNAs related to circUCK2 was determined by RT-PCR after knocking down circUCK2 in EnzS-C4-2 cells. (B) According to the prediction of Bioinformatics tools (miRBase, circRBase, and RNA22), circUCK2 has binding sites with miR-767-5p. (C) The miR-767-5p can physically interact with circUCK2. The biotinylated oligo that is complementary to the junction region of circUCK2 is mixed with the EnzR-C4-2 cell lysate, followed by purification with streptavidin beads. miR-767-5p was quantified in the pull-down complex as well as the control complex without the biotinylated oligo. (D-E) MTT assay shows that cell proliferation (D) and invasion (E) inhibition effect by overexpressing circUCK2 can be reversed by overexpressing miR-767-5p in EnzR-C4-2 cells. The data shown represent the mean of three independent experiments. *p < 0.05 by Student’s t-test for two groups or ANOVA for more than two groups.

To detect whether circUCK2 can regulate the expression of miRNAs, we screened 72 circUCK2-related miRNAs and did not find a significant increase in miRNAs when we knocked down circUCK2 in EnzS-C4-2 cells (Fig. 2A). Moreover, predictive analysis showed that circUCK2 has binding sites of miR-767-5p (Fig. 2B), suggesting a vital role of circUCK2 through the miRNA sponge. Then, the pull-down assay using the biotinylated oligo was employed to examine the interaction of circUCK2 with miR-767-5p in EnzR-C4-2 cells, and the results revealed that circUCK2 could interact with miR-767-5p (Fig. 2C). To verify that circUCK2 inhibits EnzR-C4-2 cells by lowering the level of miR-767-5p, we co-overexpressed circUCK2 and miR-767-5p. The MTT assay showed that miR-767-5p could reverse the inhibitory effect of circUCK2 on cell proliferation (Fig. 2D) and cell invasion (Fig. 2E).

Together, these results suggested that circUCK2 can sponge the miR-767-5p to reduce the proliferation and invasion ability of EnzR-C4-2 cells.

### 3.3 Mechanism dissection of how circUCK2 can suppress the PCa cell growth by increasing the TET1 expression

**Figure 3:**
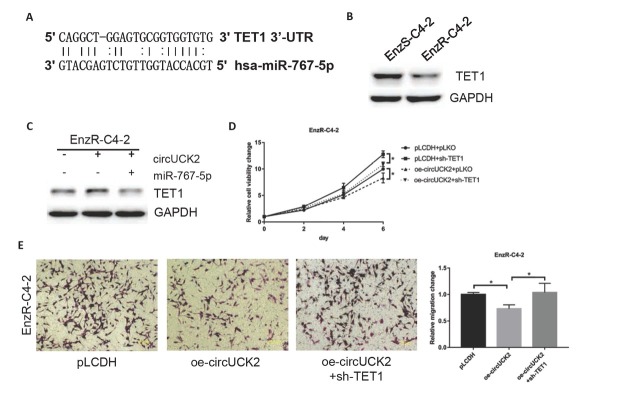
Mechanism dissection of how circUCK2 can suppress the PCa cell growth: via increase TET1 expression. (A) After searching the literature and database, we found that miR-767-5p can interact with 3’-UTR of TET1. (B) West blotting shows that the expression of TET1 was decreased in EnzR-C4-2 cells. (C) West blotting shows that the level of TET1 was increased by overexpressing circUCK2, which can be reversed by overexpressing miR-767-5p in EnzR-C4-2 cells. (D-E) MTT assay shows that cell proliferation (D) and invasion (E) inhibition by overexpressing circUCK2 can be reversed by knocking down TET1 in EnzR-C4-2 cells. The data shown represent the mean of three independent experiments. *p < 0.05 by Student’s t-test for two groups or ANOVA for more than two groups.

To understand the mechanism underlying the miR-767-5p-regulated cell proliferation and invasion, we examined the literature and bioinformatics tools (miRbase, Target-scan, and RNA22) and found that miR-767-5p can interact with the 3’-UTR of TET1 (Fig. 3A). Moreover, the expression of TET1 was decreased in EnzR-C4-2 cells (Fig. 3B). TET1 was also increased when circUCK2 was overexpressed and could be reversed by overexpressing miR-767-5p in EnzR-C4-2 cells (Fig. 3C). Furthermore, we knocked down TET1 in EnzR cells overexpressing circUCK2 and found that the cell proliferation was increased (Fig. 3D). In addition, the cell invasion could be reversed by knocking down TET1 in EnzR-C4-2 cells (Fig. 3E).

**Table 1:** Primer sequence for RT-PCR

primer	sequence
TET1 F	CATCAGTCAAGACTTTAAGCCCT
TET1 R	CGGGTGGTTTAGGTTCTGTTT
GAPDH F	GGAGCGAGATCCCTCCAAAAT
GAPDH R	GGCTGTTGTCATACTTCTCATGG
circUCK2 F	AGAGACAGAGGAGGGTCTTC
circUCK2 R	TTCTGCTCCGAGGTAAGGAC

**Table 2:** Insertion sequence for plasmids

plasmid	vector	insert sequence
oe-miR-767-5p	pLVX-ZsGreen-miRNA-Puro	TGCACCATGGTTGTCTGAGCATG
oe-circUCK2	pLCDH-ciR	TCTTCCGTGTGTGCTAAGATCGTGCAGCTCCTGGGGCAGAATGAGGTGGACTATCGC-CAGAAGCAGGTGGTCATCCTGAGCCAGGATAGCTTCTACCGTGTCCTTACCTCGGAG-CAGAAGGCCAAAGCCCTGAAGGGCCAGTTCAACTTTGACCACCCGGATGCCTTTGACAAT-GAACTCATTCTCAAAACACTCAAAGAAATCACTGAAGGGAAAACAGTCCAGATCCCCGT-GTATGACTTTGTCTCCCATTCCCGGAAGGAGGAGACAGTTACTGTCTATCCCGCAGACGT-GGTGCTCTTTGAAGGGATCCTGGCCTTCTACTCCCAGGAGGTACGAGACCTGTTCCAGAT-GAAGCTTTTTGTGGATACAGATGCGGACACCCGGCTCTCACGCAGAGTATTAAGGGA-CATCAGCGAGAGAGGCAGGGATCTTGAGCAGATTTTATCTCAGTACATTACGTTCGT-CAAGCCTGCCTTTGAGGAATTCTGCTTGCCAACAAAGAAGTATGCTGATGTGATCATCCCTA-GAGGTGCAGATAATCTGGTGGCCATCAACCTCATCGTGCAGCACATCCAGGACATCCT-GAATGGAGGGCCCTCCAAACGGCAGACCAATGGCTGTCTCAACGGCTACACCCCT-TCACGCAAGAGGCAGGCATCGGAGTCCAGCAGCAGGCCGCATTGACCCGTCTCCATCG-GACCCCAGCCCCTATCTCCAAGAGACAGAGGAGGG
sh-TET1	pLKO.1	CCGGGCAGCTAATGAAGGTCCAGAACTCGAGTTCTGGACCTTCATTAGCTGCTTTTTG

Together, these results Fig. 3A–E suggested that circUCK2 inhibits cell proliferation and invasion by increasing the level of TET1.

### 3.4 Preclinical study using the mouse model to prove that circUCK2 can inhibit EnzR cell growth

**Figure 4:**
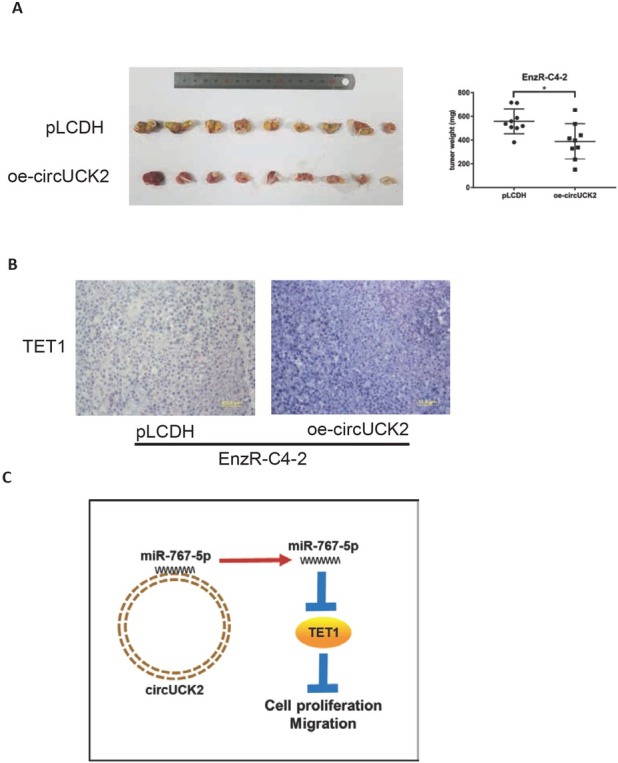
Preclinical study using the mouse model to prove that circUCK2 can inhibit the EnzR cell growth. (A) EnzR-C4-2 cells were transfected with pLCDH and circUCK2 and implanted into NDO/SCID mice. After 8 weeks, the tumour weights of the xenografts were tabulated and shown in dot plot. (B) The expression of TET1 in the two groups of mice. (C) Schematic of the circUCK2 pathway. The data shown represent the mean of 9 NDO/SCID mice in each group. *p < 0.05 by Student’s t-test for two groups.

To elucidate whether the high expression of circUCK2 gene can slow down the cell proliferation in vivo experiments, NOD/SCID mice were transplanted with EnzR-C4-2 pLCDH cells and EnzR-C4-2 oe-circUCK2 cells in situ. After 8 weeks, the EnzR-C4-2 circUCK2 group was found to have a smaller tumour size than the control group (Fig. 4A). IHC showed an increased expression of tumour TET1 protein in the EnzR-C4-2 circUCK2 group (Fig. 4B).

Together, the in vivo results showed that high circUCK2 expression inhibits the EnzR-C4-2 cell growth.

## 4 Discussion

PCa cancer is one of the most common malignancies in men[18]. Androgen deprivation is the standard first-line treatment for metastatic progressive PCa, which includes both drug and surgical castration, and a majority of these patients would develop castration-resistant prostate cancer (CRPC) within 2-3 years [19]. Enz, as a second-line drug, is more effective than only castration in inhibiting the level of AR. However, previous studies have shown that after EnzR, PCa cells continue to grow with the activation of other signalling pathways with a robust ability to metastasize and invade[20].

In the present study, after the inactivation of AR, cell growth and metastasis were studied from the perspective of circRNAs. Recent studies have shown that circRNAs are abundant in miRNA binding sites, which play a role in the sponge adsorption of miRNA in cells and block or reduce the inhibitory effect of miRNA on genes, thereby promoting the expression of target genes. Analysis of a subset of miRNAs suggests that miRNAs are involved in several important processes in life processes, including early development, cell proliferation, apoptosis, cell death, fat metabolism, cell differentiation and cancer [21, 22, 23]. This mechanism is known as the competitive endogenous RNA mechanism [24, 25]. CircRNAs also have sponge adsorption on extracellular miRNA, which decreases the activity of miRNA, and thus, destroys the immune response [26]. Consecutively, the miRNA can regulate its stability by binding to circRNAs to achieve the regulation of the miRNA level. Recently, accumulating evidence has demonstrated that abnormal circRNAs expression is related to tumour formation, malignant proliferation, and metastasis [27].

We speculated that circUCK2 plays a role with miRNAs sponge. After the adsorption of miR-767-5p, the level of free miRNA decreases, and the level of the downstream protein increases. Loriot et al. also found that miR-767 inhibited the expression of TET1/3 mRNA and protein [28]. Therefore, herein, we did not focus on the mechanism underlying miR-767-5p-mediated regulation of TET1 but mainly focused on whether circUCK2 plays a role via miR-767-5p and TET1. In the current study of prostate cancer cells, after knocking down circUCK2 EnzS type, cell proliferation and invasion were increased, but the low expression of circUCK2 EnzR PCa showed that only the invasive ability increases, while the proliferation is not promoted. Furthermore, we speculated that cell proliferation and invasion are regulated by a broad network of cellular signalling pathways [29, 30, 31, 32]. Takayama et al. demonstrated that the RANK/RANKL signalling pathway increases the risk of bone metastasis in PCa under castration [5]. Kallio et al. and our previous study showed that the expression of AR-v7 increased significantly after EnzR, thereby promoting cell progression [33, 34]. Therefore, it can be deduced that circRNAs also play a critical regulatory role in castration and EnzR in PCa cells, as well as in the progression of prostate cancer. In future clinical treatment, the expression of TET1 can be detected after the patient enters the EnzR stage, and further treatment can be assisted by TET1 agonists or the elevated expression of TET1.

In summary, the current study reveals a novel mechanism of the growth and invasion of EnzR-PCa cells. These mechanistic insights can help to prevent the PCa development in EnzR-PCa patients based on circUCK2 and TET1 expression. Nevertheless, this study did not investigate how Enz decreased the expression of circUCK2 after resistance, and further studies would be essential on the formation and regulatory mechanism of circUCK2 by AR.
